# Safety and Effectiveness of Limited Incision Facelifts: A Systematic Review and Meta-analysis

**DOI:** 10.1093/asjof/ojad027.003

**Published:** 2023-04-14

**Authors:** Ricardo O Amador, Ryoko Hamaguchi, Richard Bartlett, Indranil Sinha

**Affiliations:** Mass General Brigham, Harvard, Boston; Mass General Brigham, Harvard, Boston; Mass General Brigham, Harvard, Boston; Mass General Brigham, Harvard, Boston

## Abstract

**Goals/Purpose:**

Limited incision facelifts offer a less invasive approach compared to a standard facelift and allow for the procedure to be completed under local anesthesia. While there have been various types of limited incision facelifts described, there have been no systematic reviews of their evidence. The purpose of this study is to systematically review the limited incision facelift literature, describe the various surgical approaches and their indications, and summarize their safety and effectiveness.

**Methods/Technique:**

A systematic review of the literature was performed following the Preferred Reporting Items for Systematic Reviews and Meta-analysis guidelines and registered with the PROSPERO international prospective register of systematic reviews. The MEDLINE (Pubmed), EMBASE, and Cochrane Central Register of Trials were queried. Screening and full-text review of studies was conducted by two independent evaluators using predetermined inclusion and exclusion criteria.

Data obtained from each eligible study included information on author specialty, Oxford Centre for Evidence-Based Medicine level of evidence, and patient demographics. Operative data included anesthetic approach, operative duration, incisional pattern, SMAS technique, concomitant procedures, and drain or tissue sealant use. Complication frequencies were calculated from those studies that specifically described complication data and included the prevalence of hematoma, wounds or skin necrosis, infection, nerve injuries, and revision surgery. Data on patient- and provider-reported outcomes were also collected.

Meta-analyses were performed using aggregated data. Proportions are provided with the corresponding 95% confidence interval. A Random-effects model was used to summarize complications data and meta-regressions were conducted to analyze associations with operative variables.

**Results/Complications:**

A total of 1137 articles were retrieved using the pre-specified search criteria. Following the removal of duplicates, 758 studies were screened using titles and/or abstracts, and 144 articles underwent full-text review. Review yielded twenty articles that met inclusion criteria. Using the Oxford Centre for Evidence-Based Medicine guidelines, 17 (85%) articles provided level IV evidence while three (15%) provided level III evidence. Articles included a total of 4451 patients with an average age of 56 years. Ninety-four percent were women while 6% were men.

Forty-two percent of patients underwent surgery awake under local anesthesia, 24% under IV sedation, 18% under intramuscular sedation, and 16% under general anesthesia. Incisional patterns varied but were categorized as limited to pre-auricular (5%), pre-and retro-auricular (10%), pre-auricular with horizontal sideburn extension (30%), pre-auricular with temporal extension (35%), and pre- plus retro-auricular with temporal extension (20%) (Figure). SMAS technique included plication (33%), purse-string suspension (28%), plication plus SMASectomy (11%), plication plus SMASectomy and purse-string suspension (11%), SMASectomy only (6%), or other (18%). Forty-five percent of articles described using drains and 10% described using a tissue sealant.

The overall complication frequency was 4.0% (95% CI 2.8-5.2%). The use of a drain or sealant was associated with a decreased complication frequency of 2.6% (95%CI 1.0-4.2%) compared to 5.0% (95% CI 3.4-6.6%) in studies using no drain or sealant (p=0.04). There were no associations found between complications and incision type or anesthetic approach. Eighteen studies described hematoma frequencies. Among these articles, there was a 2.3% hematoma frequency (95% CI 1.5-3.0%). Twenty-seven patients (1.5%) had hematomas that were treated with conservative management like needle aspiration while eight (0.4%) required a return to the operating room.

The next most common complication described was temporary nerve injury or neuropraxia with a rate of 0.2% (95% CI 0.1%-0.4%­­­). The frequency of skin necrosis or non-healing wounds was 0.2% (95% CI 0.1%-0.04%). No permanent nerve injuries were described. Other less commonly described complications included widening or hypertrophic scars, dog ear deformities (all within patients undergoing pre-auricular limited incisions), skin dimpling, three cases of parotid fistula, and a DVT in a patient undergoing multiple concomitant body procedures. Seven articles (35%) provided data on patient-reported outcomes while three (15%) provided data on provider-based outcomes, such as ratings of postoperative results.

**Conclusion:**

To our knowledge, this is the first systematic review and meta-analysis of limited incision facelifts. This surgical approach offers an alternative to traditional rhytidectomy for the properly selected candidate. Meta-analysis demonstrates a low postoperative complication rate which is further reduced in studies using a drain or tissue sealant. This meta-analysis provides plastic surgeons and their patients with an evidence-based reference for complication data and can improve the preoperative counseling process. Future studies should focus on incorporating validated patient-reported outcome measures and comparing surgical approaches.

